# The Sigma-trial protocol: a prospective double-blind multi-centre comparison of laparoscopic versus open elective sigmoid resection in patients with symptomatic diverticulitis

**DOI:** 10.1186/1471-2482-7-16

**Published:** 2007-08-03

**Authors:** Bastiaan R Klarenbeek, Alexander AFA Veenhof, Elly SM de Lange, Willem A Bemelman, Roberto Bergamaschi, Piet Heres, Antonio M Lacy, Wim T van den Broek, Donald L van der Peet, Miguel A Cuesta

**Affiliations:** 1 Department of Surgery, VU medical centre, Amsterdam, The Netherlands; 2 Department of Clinical Epidemiology and Biostatistics, VU medical centre, Amsterdam, The Netherlands; 3 Department of Surgery, Academisch Medisch Centrum, Amsterdam, The Netherlands; 4 Department of Surgery, Central Teaching Hospital, Bergen University, Forde, Norway; 5 Department of Surgery, Waterland Hospital, Purmerend, The Netherlands; 6 Department of Surgery, Hospital Clinic, Barcelona, Spain

## Abstract

**Backround:**

Diverticulosis is a common disease in the western society with an incidence of 33–66%. 10–25% of these patients will develop diverticulitis. In order to prevent a high-risk acute operation it is advised to perform elective sigmoid resection after two episodes of diverticulitis in the elderly patient or after one episode in the younger (< 50 years) patient. Open sigmoid resection is still the gold standard, but laparoscopic colon resections seem to have certain advantages over open procedures. On the other hand, a double blind investigation has never been performed. The Sigma-trial is designed to evaluate the presumed advantages of laparoscopic over open sigmoid resections in patients with symptomatic diverticulitis.

**Method:**

Indication for elective resection is one episode of diverticulitis in patients < 50 years and two episodes in patient > 50 years or in case of progressive abdominal complaints due to strictures caused by a previous episode of diverticulits. The diagnosis is confirmed by CT-scan, barium enema and/or coloscopy.

It is required that the participating surgeons have performed at least 15 laparoscopic and open sigmoid resections. Open resection is performed by median laparotomy, laparoscopic resection is approached by 4 or 5 cannula. Sigmoid and colon which contain serosal changes or induration are removed and a tension free anastomosis is created. After completion of either surgical procedure an opaque dressing will be used, covering from 10 cm above the umbilicus to the pubic bone. Surgery details will be kept separate from the patient's notes.

Primary endpoints are the postoperative morbidity and mortality. We divided morbidity in minor (e.g. wound infection), major (e.g. anastomotic leakage) and late (e.g. incisional hernias) complications, data will be collected during hospital stay and after six weeks and six months postoperative. Secondary endpoints are the operative and the postoperative recovery data. Operative data include duration of the operation, blood loss and conversion to laparotomy. Post operative recovery consists of return to normal diet, pain, analgesics, general health (SF-36 questionnaire) and duration of hospital stay.

**Discussion:**

The Sigma-trial is a prospective, multi-center, double-blind, randomized study to define the role of laparoscopic sigmoid resection in patients with symptomatic diverticulitis.

## Background

Diverticulosis is a common disease in the western society with an overall incidence of 33% in the population > 45 years of age and increasing to 66% in the population older than 85 years of age [1,2]. 10–25% of patients with diverticulosis will develop diverticulitis [3,4]. Treatment of diverticulitis is based on the severity of the disease, which can be staged by the Hinchey criteria, shown in table 1[5-8]. Most patients with stage I and II disease can be treated conservatively with bed rest, starvation, intra venous fluids and intra venous antibiotics. Abscesses greater than 5 cm are drained percutanously. In case of clinical decline of the patient or patients with stage III and IV disease, acute surgical intervention is indicated [5-8]. The inflamed bowel, mostly the sigmoid, has to be resected with or without creating a primary anastomosis. Mortality in these patients is relatively high, 6 to 22%, due to the severity of the disease [8].

**Table 1:** Hinchey stage for diverticulitis.

I	Acute phlegmonous diverticulitis without complications
II	Diverticulitis with parcolic abscess without perforation
III	Diffuse purulent peritonitis
IV	Diffuse faecal peritonitis

The chance of developing a second episode of diverticulitis within five years after a successfully conservative treatment is 30% [6,9]. In order to prevent a high-risk acute operation it is therefore advised to perform an elective (sigmoid) resection of the affected bowel after two episodes of conservatively treated diverticulitis in the elderly patient or after one episode in the younger (< 50 years) patient. When the acute phase of the diverticulitis has calmed the resection can be performed, which will mostly take place after 3 months, resulting in a mortality rate of 2%.

Also patients with complaints such as pain, recurrent bleeding and important change of bowel movements due to stenosis of the sigmoid after an episode of diverticulitis may require an elective resection of the sigmoid.

Resection of the sigmoid by an open procedure is still the gold standard but it is also feasible to perform a laparoscopic sigmoid resection [10-14].

Because laparoscopic colon resections seem to have important short term advantages (less complications, less post-operative pain, less wound infections, shorter hospital stay and faster recovery) over an open procedure, the aim of this study is to compare, in terms of post-operative morbidity, mortality and quality of life, the laparoscopic resection of the sigmoid with an open resection in those patients in which an indication exist for an elective resection [15-18].

## Method

### Study objectives

The objective of this study is to define the role of laparoscopic treatment in patients with symptomatic diverticulitis. The Sigma-trial is a prospective, multi-center, double-blind, randomized study. Patients with symptomatic diverticulitis are randomized for either open or laparoscopic sigmoid resection. Our hypothesis is that patients undergoing laparoscopic sigmoid resection have fewer complications, less pain and earlier discharge from the hospital.

### Endpoints

Primary endpoints of the study are postoperative morbidity and mortality. Minor complications are defined as wound infections, urine tract infections pneumonia, venous thrombosis or other. Major complications consist of anastomotic leakages, postoperative haemorrhage, intra-abdominal abscesses, re-operations and other within the first four postoperative weeks. Data concerning late complications after four weeks are collected at the outpatient clinic six months postoperative (i.e. incisional hernia, ileus or other).

The secondary endpoints are procedure related events such as duration of operation, conversion to open procedure in laparoscopic group, blood-loss during operation, number of re-admissions and re-operations. Furthermore postoperative recovery data are hospital stay (days), type and number of analgesics needed after operation, VAS-painscore, return to fluid and normal diet, postoperative ileus, SF-36 questionnaire (general health) translated and adapted to nationality, recurrence rate after five years.

### Power of the study

According to the published literature and own experience at the VU medical centre a difference in morbidity of 23% is found between the open procedure (35%) and the laparoscopic procedure (12%) [19-21]. To demonstrate this difference of 23%, using α = 0.05 and β = 0.2, two groups of 52 patients are required. This means that a total of 104 patients have to be included.

### Inclusion criteria

Patients who were admitted for a conservatively treated episode of diverticulitis, who will therefore undergo an elective resection of the sigmoid. The indication for elective resection is in patients < 50 years after one episode of conservatively treated diverticulitis and in patients older than 50 years after two episodes of diverticulitis or in case of progressive abdominal complaints due to strictures caused by a previous episode of diverticulits. Moreover patients with recurrence of bleeding are candidates for inclusion. The diagnosis diverticulitis is confirmed by CT-scan and/or barium enema and coloscopy.

Operation will take place at least after three months of the last attack of diverticulitis.

### Exclusion criteria

Exclusion criteria are signs of acute diverticulitis, previous infra umbilical laparotomy or previous colorectal surgery or no informed consent. Presence of a fistula is not considered as exclusion criteria.

### Participating surgeons and clinics

Complication rate, duration of operation and morbidity can be a result of the learning curve of the operating surgeon. To prevent surgeon bias, the open and laparoscopic operations have to be performed by surgeons experienced in both techniques. It is reported that after 15 operations, operating time decreases significantly which could be an indication of the end of the learning curve [22]. Therefore it is required that the participating surgeon has performed at least 15 laparoscopic sigmoid resections as well as 15 open sigmoid resections.

Five European academic and non-academic centres will participate in the study: Academisch Medisch Centrum, Amsterdam; Hospital Clinic, Barcelona; Central teaching Hospital, Forde; Waterland Ziekenhuis, Purmerend; and VU medical centre, Amsterdam.

### Randomization and blinding

The patient will be informed about the trial at the outpatient clinic. When informed consent is obtained, the patient will be randomised at the outpatient clinic. Randomization is performed by an internet randomization module. Participating surgeons are allowed to login to the secured Sigma-trial website, after filling out the randomization form immediate response with randomization number and type of operation is obtained.

In order to perform a double-blind study, the patient will not be informed which procedure he or she will undergo. Also the ward physician and nurses are not informed about the type of procedure. Patients will be put on the waiting list as indication: 'Sigma-trial', without verifying the type of operation. Postoperative an opaque dressing will be applied to cover the wounds for five days and the surgery details will be kept separate from the patient's notes. When the patient does not agree to participate he or she will undergo the standard procedure in the corresponding department.

### Data collection and statistics

Data are partially collected via a secured internet module, which is specially designed for the Sigma-trial, and via datasheets on paper, which are sent to the VU medical centre by mail. Data are collected daily until the day of discharge. Preoperatively, at six weeks and six months postoperatively the SF-36 questionnaire is filled in by the patient.

There will be regular contact between the study coordinators and the participating centres. One research fellow will monitor the data of every included patient. An SPSS-database will be created with all required parameters. Data analysis will be performed in accordance with the intention to treat principle. Groups will be compared using an Independent Samples T-test where appropriate, other wise a Willcoxon or Chi-square tests. Painscores will be analysed using repeated measures analysis.

### Ethics

This study is conducted in accordance with the principles of the Declaration of Helsinki and 'good clinical practice' guidelines. The independent medical ethics committees of the participating hospitals have approved the study protocol. Prior to randomization, written informed consent will be obtained from all patients.

## Surgical technique

### Preoperative preparation

Both patients who will undergo laparoscopic and open resections will receive standard bowel preparation. Before operation both groups will receive standard prophylactic antibiotics: Cefuroxim 1500 mg and Metronidazol 500 mg.

### Open resection

A median laparotomy with resection of macroscopically bowel with diverticulosis will be performed. Proximal resection margin is defined by resection of the sigmoid and colon which contains serosal changes of prior inflammation or induration of the colonic mesentery. It is more important to resect this colonic segment then to remove all present diverticula's. It is not always necessary to mobilize the splenic flexure in order to create a tension free anastomosis. The distal margin is made to the anatomic rectum, which can be identified by the loss of the taeniae coli. No diverticulae should be placed in the anastomosis. Usually a double-stapled anastomosis is created.

### Laparoscopic resection

**Figure 1:**
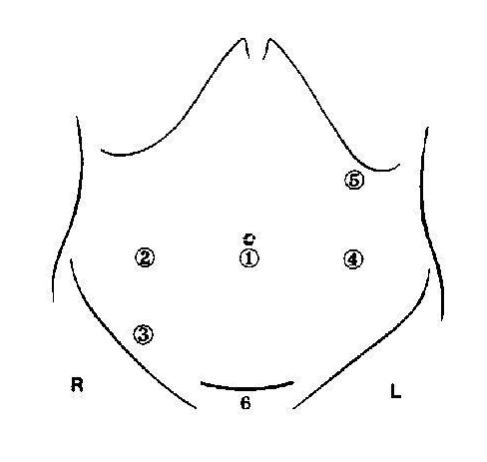
Trocart postion for laparoscopic sigmoid resection. Trocart 5 is optional: when mobilization of splencic flexure is required. 6 = pfannenstiel incision for retrieval of specimen.

Depending on whether mobilization of the splenic flexure is necessary, a four or five cannula approach will be used, shown in figure 1. In Trendelenburg position, sigmoid dissection is performed medial to lateral if possible. First of all the sigmoid vessels are being dissected and cut using endostaplers or devices such as Ultracision (Johnson & Johnson Gateway) or Ligisure (Valleylab, Tyco). After mobilisation of the mesenterium up to the descending colon and presacral (with preserving of the hypogastric nerves), the sigmoid is mobilised. In the case of extensive diverticulitis or small loop of the sigmoid the splenic flexure is mobilized. At the level of the promotory the mesorectum is skeletized and the bowel will be cut using an endostapler. A Pfannenstiel incision is made, the specimen is retrieved and the resection is performed. Then the anvil of the circular stapler is introduced. After reintroduction in the abdomen and closure of the wound and reinsufflation a double-stapled tension-free anastomosis is created.

After completion of surgery, all details are recorded in a trial folder, which will be kept separate from the patient's notes. This will be handed over to the nurse in charge of the ward and access to it will be restricted to emergency situations only.

### Postoperative management

**Figure 2:**
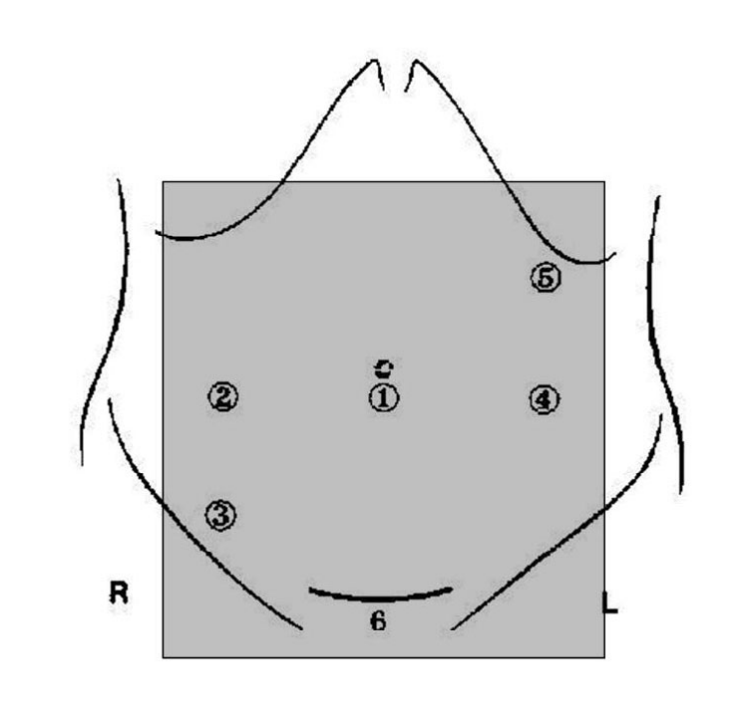
Placement of dressing.

In both procedures an identical opaque dressing, covering from 10 cm above the umbilicus to the pubic bone, as shown in figure 2, will be used irrespective of which operation is performed. To avoid guessing by the nurses and ward physician about what type of operation is used the dressing will be stained with bloodstained fluid or aqueous iodine. The dressing may not be disturbed unless there is a problem (wound pain, fever). In that case it will be removed for examination of the wound. In case of early removal of the dressing, the routine ward physician and routine nurse may not be informed which type of operation was performed. The dressing will routinely be removed at day five postoperative or earlier if the patient is dismissed before day five. As a control of the blinding method, at day two the patient will be asked which type of operation he or she underwent.

The principles of the fast track protocol for major colonic and rectal surgery as described by Delaney et al, will be applied [23]. When a drain is placed it will be removed one day after surgery. Postoperative pain will be treated by "Patient Controlled Analgesia" intravenously (PCA), a pump with morphine until maximal 72 hours after the operation. Patient will be advised by the anaesthesiologist about the PCA pump. Morphine doses will be noted. Oral analgesia (Paracetamol: 4 times 1 gram/24 hour) is started two days after the operation. Bladder catheter will be removed the second day after the operation. The nasogastric tube will be removed before endotracheal extubation. Non-carbonated liquids are offered the evening after the operation. Patient can decide when he or she wants to extend the diet to blend and solid food when oral fluids are tolerated.

To regain early mobilisation, patients are encouraged to sit out of bed starting one day after the operation. Patient will be discharged when they pass stool, are able to drink, eat solid food, can walk and are comfortable with oral analgesia. Delay due to "social" reasons will be noted. An independent physician will decide when the patient is going to be discharged.

Follow-up is carried out at the outpatient clinic, patients will be routinely seen at six weeks and six months after the operation.

## Discussion

Laparoscopic colon surgery is being successfully performed since 1991 [24]. During the last decade several prospective series of patients and comparative studies between open and laparoscopic approach of a group of patients with diverticulitis have been published. Short term advantages of laparoscopic over open treatment for elective sigmoid resection for symptomatic diverticulitis, including less operative trauma, decreased postoperative pain, early discharge from the hospital and less morbidity [25-32]. Differences between 35% of morbidity in the open procedure versus 12% for the laparoscopic approach have been published [19-21].

Despite the feasibility of the procedure, still only a small percentage of all elective sigmoid resections are done laparoscopically. Possibly the disturbed anatomy, due to recurrent inflammation, makes laparoscopic dissection a difficult technical challenge. Other disadvantages might be increased operating time, high conversion percentage to laparotomy and higher costs.

A prospective, randomized study comparing the laparoscopic versus the open approach has been considered necessary in order to answer these questions.

## Abbreviations

VAS-painscore: Visual Analogue Scale Painscore

SF-36: Short Form-36

## Competing interests

The author(s) declare that they have no competing interests.

## Authors' contributions

BRK drafted the manuscript. MAC co-authored the writing of the manuscript. AAFAV and DLP contributed in the data collection. All other authors participated in the design of the study during several meetings and are local investigators at the participating centres.

All authors edited the manuscript and read and approved the final manuscript.

## Pre-publication history

The pre-publication history for this paper can be accessed here:


